# Digital Recordings of Centric Relation Using Conventional and Digital Techniques: Patient-Reported Outcome Measures (PROMs)

**DOI:** 10.3390/jcm15062232

**Published:** 2026-03-15

**Authors:** Ece Selen Koçar, Kıvanç Akça

**Affiliations:** Department of Prosthodontics, Faculty of Dentistry, Hacettepe University, 06230 Ankara, Türkiye

**Keywords:** PROMs, jaw relation, centric relation, jaw-tracking device, bimanual manipulation

## Abstract

**Background/Objectives:** Centric relation (CR) is a reproducible mandibular reference position that plays a critical role in complex prosthodontic cases. With the advent of digital jaw-tracking devices, CR can now be recorded with greater precision through fully digital methods. This study aimed to compare patient-reported outcome measures (PROMs) for the recording of CR determined with conventional and digital techniques. **Methods:** Patients requiring occlusal rehabilitation due to bilateral loss of posterior support in the maxilla were included. Two different jaw relation recording techniques were applied: conventionally determined CR and digitally determined CR. The former was determined using bimanual manipulation, while the latter through multiple mandibular closure recordings performed with an anterior plateau using a jaw-tracking device. PROMs were assessed using Visual Analog Scale (VAS) to evaluate patient experience during jaw relation recording and comfort during restoration try-in. The recording time for both techniques was documented, and the correlation between recording time and VAS scores related to the recording procedure was analyzed. Statistical analyses were performed using the Wilcoxon signed-rank test and Spearman correlation analysis (α = 0.05). **Results:** Twelve patients were included. No statistically significant difference was found between the two methods in VAS scores assessing patient-reported comfort and experience. Recording time was significantly shorter for the recording of conventionally determined CR (*p* = 0.002). No statistically significant correlation was found between recording time and patient-reported experience for both techniques (*p* > 0.05). **Conclusions:** Despite the need for clinician experience and patient compliance, PROMs for digitally determined CR were comparable to those of conventionally determined CR.

## 1. Introduction

The primary load-bearing components of the masticatory system include posterior teeth and their supporting alveolar bone, temporomandibular joint (TMJ), and anterior teeth. The majority of occlusal support is provided by the posterior teeth and is referred to as posterior support. Loss of posterior teeth may result in impaired masticatory function, esthetic deficiencies, and most importantly, changes in the occlusal vertical dimension (OVD) [1]. OVD is a fundamental parameter in maintaining the neuromuscular (NM) balance of the masticatory system, and failure to reestablish it accurately may lead to adverse effects on the TMJ and the NM system. Therefore, the accurate and predictable reconstruction of OVD in patients with posterior support loss represents a critical step in prosthetic treatment planning [2].

The accurate recording of the jaw relation is one of the fundamental requirements for achieving biologically and mechanically successful prosthetic restorations, particularly in complex cases such as occlusal rehabilitation. Centric relation (CR), defined as a repeatable and clinically stable reference position of the mandible, is widely recommended for full-arch restorations, cases requiring changes in the OVD, and occlusal rehabilitations [3,4,5]. Accurate determination of CR and its proper transfer to restorative procedures are crucial for long-term functional stability and esthetic predictability. The inaccurate determination or transfer of CR and OVD has been associated with TMJ disorders and prosthetic complications. With the increasing use of monolithic zirconia restorations, inappropriate occlusal relationships may result in uneven force distribution and localized stress accumulation, leading to cracks, chipping, or catastrophic failures [6,7,8,9,10]. Recent evidence [11,12,13] suggests that digital approaches enhance the accuracy and reproducibility of restorative procedures, thereby supporting the growing use of digital jaw relation recording methods to help prevent associated clinical complications [14,15,16].

Methods for the determination of CR have been classified in the literature in various ways and are generally described as physiologic, guided, and NM techniques [17,18]. Although differing opinions exist regarding the accuracy and repeatability of these methods, guided techniques have been reported to provide high repeatability when performed by experienced clinicians [19,20,21]. Nevertheless, the reliability of CR determination techniques is highly dependent on clinician experience and patient cooperation, and their relative superiority remains debatable. Axiographic systems allow for the functional assessment of mandibular movements and the dynamic analysis of condylar pathways, thereby contributing to the identification and evaluation of CR as a reproducible mandibular reference position [22,23,24,25]. However, the clinical application of axiographic systems is often considered technique-sensitive and time-consuming, which has limited their routine use and encouraged the adoption of more simplified clinical approaches. In recent years, with the increasing prevalence of digital workflows in prosthodontics, the use of jaw-tracking devices for obtaining and recording jaw relation has increased.

In clinical procedures that require direct patient cooperation, such as jaw relation recording, both the technical accuracy of the method and the patient’s perceived comfort and procedural experience may influence the treatment process. Differences in patient experience between digital and conventional methods have been reported in the literature [26,27,28,29]. In this context, PROMs are regarded as essential assessment tools that complement clinical parameters and incorporate the patient’s perspective into the treatment process [30,31,32]. By focusing on outcomes that are most meaningful to patients, PROMs enable the evaluation of clinical procedures beyond purely technical or clinician-centered measures [33].

Because PROMs directly reflect patient experience, they offer important advantages in evaluating subjective yet clinically meaningful outcomes such as comfort, tolerance, and procedural experience. The current literature indicates that the use of PROMs allows for the quantitative assessment of the patient’s perceived comfort and procedural experience beyond the technical outcomes of clinical procedures, thereby contributing to patient-centered clinical evaluation [34,35,36].

Subjective assessment tools such as the Visual Analog Scale (VAS) allow the quantitative recording of patient experiences and enables clinicians to better balance technical accuracy with patient satisfaction. Accordingly, patient-reported outcomes are increasingly used in the evaluation of jaw relation recording methods, not only to assess patient satisfaction but also to support patient-centered treatment planning [31,32,37].

However, no clinical studies have evaluated the use of jaw-tracking devices in occlusal rehabilitation cases from a PROM perspective. Therefore, the aim of this study was to compare the recordings of conventionally determined centric relation (c-CR) and digitally determined centric relation (d-CR) in patients with posterior support loss requiring occlusal rehabilitation, based on PROMs.

Accordingly, the primary null hypothesis of the study was that there would be no difference in patient-reported outcomes, specifically clinical procedure experience and comfort, between recordings of c-CR and d-CR. The secondary null hypothesis was that there would be no correlation between recording time and patient-reported experience during the jaw relation recording procedure.

## 2. Materials and Methods

### 2.1. Study Group

This study included individuals aged 40–55 years who presented to the Faculty of Dentistry, Hacettepe University, for treatment and required bilateral posterior fixed prosthetic treatment and occlusal rehabilitation in the maxilla. The study protocol was approved by the Hacettepe University Clinical Research Ethics Committee (Decision No.: 2024/18-07; Approval Code: KA-24002) and was registered at ClinicalTrials.gov (Identifier: NCT06982898). Written informed consent was obtained from all participants prior to enrollment.

Inclusion criteria:

1. Age between 40 and 55 years;
2. Presence of healthy periodontal tissues and TMJ [38,39];
3. Bilateral loss of occlusal support involving two premolars and two molars in the maxilla;
4. Indication for single-tooth crowns or/and terminal-supported fixed dental prostheses;
5. Suitable abutment teeth characteristics (absence of periapical pathology, crown-to-root ratio ≥ 1:1, mobility grade I, probing depth ≤ 4 mm, vital or endodontically treated non-vital teeth) [40];
6. Presence of a fixed mandibular dentition with a physiologic occlusal plane (within the limits of the curves of Spee and Wilson) [41];
7. No contraindications to dental treatment.

Exclusion Criteria:

1. Refusal to sign the informed consent form;
2. Use of removable prostheses in the mandible;
3. Presence of temporomandibular joint dysfunction, orofacial pain, or acute oral disease;
4. History of cervical muscle disorders, cervical disk herniation, or benign paroxysmal positional vertigo.

### 2.2. Jaw Relation Determination and Recording

In all participants, tooth preparations were completed in a single session during the initial phase of treatment and were performed in accordance with previously described preparation protocols [42,43]. Following this, an intraoral scanner (TRIOS 5; 3Shape A/S, Copenhagen, Denmark) and its corresponding software (3Shape Trios 19.2.0; 3Shape A/S) were used for direct digital recording of the dental arches, and chairside provisional restorations were fabricated using polymethyl methacrylate (PMMA).

At subsequent clinical visits, jaw relations were obtained using two different methods. In the first method, CR was determined via bimanual manipulation and recorded using an intraoral scanner. In the second method, CR was digitally determined from multiple closure recordings obtained with an anterior plateau using a jaw-tracking device. The two jaw relation recording procedures were performed on two separate clinical visits, scheduled two days apart.

**Recording of Conventionally Determined Centric Relation (c-CR):**

Following completion of the preparations, the occlusal vertical dimension was determined during the second clinical session by considering functional and esthetic determinants. For myofascial relaxation, participants were instructed to perform 10–15 opening and closing movements without tooth contact [44].

During bimanual manipulation, participants were positioned in a fully supine position in the dental unit, with the head stabilized between the clinician’s torso and forearm. This positioning allowed for the controlled guidance of the mandible and prevented involuntary head movements. The clinician placed their thumbs on the patient’s chin and the remaining fingers along the inferior border of the mandible, guiding the mandible gently and continuously in a hinge movement. No sudden or excessive force was applied during manipulation, and the condyles were allowed to sit naturally within the fossae. If muscle response or resistance was perceived during mandibular movement, the procedure was discontinued and repeated after achieving muscle relaxation. When the condyles were perceived to be fully seated in the fossae and the mandible could open and close freely in a hinge movement, this position was accepted as CR [15].

**Figure 1:**
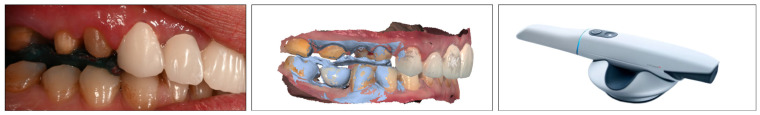
Stabilization of the centric relation (CR) determined via bimanual manipulation (**left**); digital jaw relation recording (**middle**) using an intraoral scanner (**right**).

To stabilize the CR position, a layered recording plate was prepared by placing aluminum foil between layers of patterned baseplate wax designed to prevent anterior contact, and the record was positioned intraorally (Figure 1). In the stabilized position, static interocclusal records were obtained from the right and left posterior segments using an intraoral scanner (TRIOS 5; 3Shape, Copenhagen, Denmark) following the manufacturer’s buccal bite scanning protocol. Automatic alignment was performed in the software, and the data were saved after post-processing.

In this group, CR was clinically determined using a conventional method, and the obtained jaw relation record was digitized. Digitalization of the CR record was achieved using an intraoral scanner, and the record was transferred to prosthetic planning within the digital workflow.

**Recording of Digitally Determined Centric Relation (d-CR):**

For jaw relation recording, a jaw-motion-tracking and analysis system (zebris JMA Optic; zebris Medical GmbH, Isny im Allgäu, Germany) along with its associated software (WINJAW+, version 4.0; zebris Medical GmbH) was used. The system operates based on optical triangulation technology, capturing mandibular movements through infrared signals emitted from a mandibular sensor and detected by cameras integrated into an electronic facebow. The system includes an electronic facebow, a lower jaw sensor, a C-bow for anatomical referencing, customized bite forks, paraocclusal attachments, and dedicated analysis software. Data acquisition was performed using the Function and Digital Occlusion modules of the WINJAW+ software in accordance with the manufacturer’s protocol.

Prior to each recording session, the system was set up according to the manufacturer’s instructions. A skull-related reference plane (Camper plane) was defined, anatomical reference points (porion and subnasal point) were registered, and sensor status was verified to ensure accurate measurement. The C-arch (condylar reference) tool was used for condylar positioning. Intraoral scans were aligned within the software using a four-point matching protocol, and the mandibular static reference position was recorded as the resting position.

Participants were positioned upright in the dental unit, and the electronic facebow was placed parallel to the interpupillary line and the sagittal plane. A customized paraocclusal attachment was used to attach the optical sensor to the mandible. For scan position alignment within the digital workflow, the CR wax record obtained during the conventional recording session was used to ensure positional synchronization between the intraoral scan and the jaw-tracking software.

**Figure 2:**
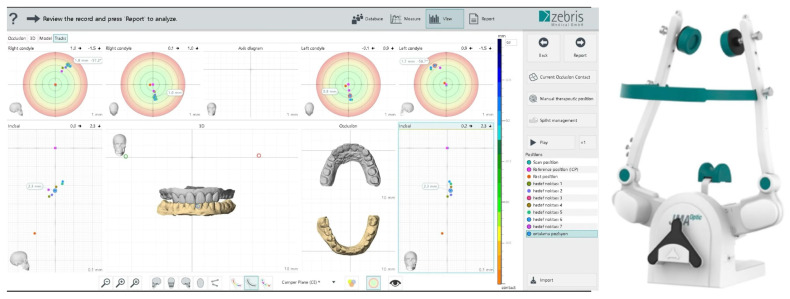
Digital determination of CR based on multiple mandibular closure recordings obtained using a jaw-tracking system: software interface illustrating the calculation of the average static mandibular position from multiple CR recordings (**left**), and the jaw-tracking device used in the study (**right**).

For myofascial relaxation, participants were instructed to perform 10–15 opening and closing movements without tooth contact [44]. A customized anterior plateau was prepared intraorally using the zebris occlusal alignment fork for maxillomandibular relation recording with the zebris JMA Optic system (jaw relation module) (Reitz, personal communication). Following preparation of the anterior plateau participants were instructed to close slowly from maximum opening to approximately 5–10 mm and subsequently perform a rapid, ballistic closing movement until contact with the anterior plateau was achieved. Each closure was recorded as a target position. This procedure was repeated seven times for each participant. The average of the XYZ coordinates of the seven target positions was calculated within the software and recorded as the “average target position” (Figure 2).

The c-CR was performed during the first session, followed by the d-CR during the second session. Randomization of the recording sequence was not applied due to the technical requirements of scan position alignment within the digital workflow. However, the digitally determined CR position was calculated independently from the averaged closure recordings and was not constrained by the conventional CR record.

In this group, CR was determined using digital methods, and the obtained jaw relation record was recorded directly in the digital environment. The determination and recording of CR were performed using a digital jaw-tracking system to obtain a static reference position from multiple closure recordings, and the record was transferred to prosthetic planning within the digital workflow.

### 2.3. PROMs

PROMs were evaluated in two clinical stages. The first evaluation was performed immediately after completion of the recording procedure and aimed to assess the experience of the recording method. The second evaluation was conducted during the try-in of the restorations fabricated based on each jaw relation record and aimed to assess the perceived comfort during restoration try-in.

After completion of the recording procedure with each method, participants were asked to complete an evaluation form. Patient experience during the clinical procedure was assessed using a Visual Analog Scale (VAS) ranging from 0 to 10, where 0 represents an extremely negative experience and 10 represents an extremely positive experience. In addition, participants who experienced both methods were asked the question, *“Which method would you prefer for the next procedure?”* Responses were recorded as a binary choice (c-CR/d-CR). All evaluations were performed immediately after participants had experienced both methods sequentially, without any operator guidance or reminders. Recording time was documented for both methods.

For the second evaluation, restorations were fabricated based on jaw relation data obtained with each recording method. The datasets were exported in appropriate formats and transferred to computer-aided design (CAD) software. All datasets were transferred to exocad software (version 3.2 Elefsina; Exocad GmbH, Darmstadt, Germany) for digital design procedures. To maintain identical occlusal morphology between the two groups, the design created for c-CR was adapted to d-CR, and final adjustments were made according to static occlusion.

The approved digital designs were manufactured using a five-axis milling system (Vhf E5; Vhf GmbH, Ammerbuch, Germany) with a subtractive manufacturing process from PMMA blocks (Polident, Volčja Draga, Slovenia). Thus, for each participant, two bilateral posterior restorations based on two different jaw relation records were obtained.

Restorations fabricated based on jaw relation records obtained with both methods were tried intraorally. During the try-in procedure proximal contacts, internal fit, and marginal adaptation were sequentially evaluated. Proximal contacts were assessed using dental floss, and internal and marginal fit were evaluated by verifying the presence of a uniform, thin film of elastomeric disclosing material (Fit Checker Advanced Blue; GC Corporation, Tokyo, Japan) under ×2.5 magnification using a dental loupe. Minor clinical adjustments were performed when necessary to achieve acceptable seating and proximal contacts, as described previously [45]. Restorations requiring major clinical adjustments or exhibiting inadequate fit were excluded from the evaluation. Following clinical approval, participants were asked to score the restorations without performing any occlusal adjustments.

During the try-in procedure, both the participant and the investigator responsible for outcome assessment were blinded, and the restorations were evaluated in a randomized order. All clinical procedures, including jaw relation recordings and try-in appointments, were performed by a single investigator (E.S.K.). Blinding procedures and the evaluation of patient-reported outcomes, including VAS assessments, were conducted by a second independent investigator (K.A.).

At the end of each try-in session, participants were asked to rate both the first-tried and second-tried restorations using a VAS based on perceived comfort. Repetitive opening and closing movements were performed between try-ins to facilitate myofascial relaxation [44].

### 2.4. Statistical Analysis

Statistical analyses were performed using IBM SPSS Statistics software (Version 21.0; IBM Corp., Armonk, NY, USA). Statistical power was calculated using G*Power 3.1 software (Heinrich-Heine-Universität Düsseldorf, Düsseldorf, Germany). As VAS scores related to jaw relation recording experience and restoration try-in comfort were considered ordinal data, non-parametric Wilcoxon signed-rank tests were used for intergroup comparisons.

Normality of the recording time variable, calculated in minutes, was evaluated using the Shapiro–Wilk test. Since the data did not show a normal distribution, the Wilcoxon signed-rank test was also applied for the comparison of recording time between the two methods.

Statistical significance was set at *p* < 0.05. Effect sizes (r) were calculated using the formula r = Z/√N where Z represents the standardized test statistic obtained from the Wilcoxon signed-rank test and N represents the total number of observations included in the comparison (N = 24). Effect sizes were interpreted according to Cohen’s criteria (small: 0.10–0.29; medium: 0.30–0.49; large: ≥0.50). In addition, Hodges–Lehmann median differences and corresponding 95% confidence intervals were reported to provide an estimate of the magnitude and precision of the observed differences.

The correlation between recording time and VAS scores related to the jaw relation recording procedure was evaluated using Spearman’s rho correlation coefficient. In addition, the association between VAS scores related to jaw relation recording experience and restoration try-in comfort was explored using Spearman’s rho correlation coefficient.

### 2.5. Sample Size Calculation

The sample size of the study was determined based on a power analysis considering findings from similar studies in the literature and the statistical methods to be used. The analysis indicated that, with a 90% statistical power (1–β = 0.90) and a 5% significance level (α = 0.05), a minimum of 13 participants would be required to detect a difference between two dependent measurements.

During the study period, 13 participants were enrolled. One participant was excluded from the analysis due to insufficient cooperation during both clinical procedures. Consequently, data from 12 participants were included in the final analysis.

A post hoc power analysis performed using G*Power 3.1 software, assuming an effect size of 0.8 and a 95% confidence level, revealed a statistical power of 81%, indicating that the final sample size was acceptable for detecting meaningful differences.

## 3. Results

A total of 12 patients were included in the study (4 males and 8 females), with a mean age of 45 years. When patient preferences regarding jaw relation determination and recording methods were evaluated, 58% of the participants preferred c-CR, whereas 42% preferred d-CR.

The mean VAS score for c-CR jaw relation recordings was 7.7 ± 1.3 (median: 7.0), whereas the mean VAS score for d-CR recordings was 7.0 ± 1.7 (median: 7.5). No statistically significant difference was observed between the two methods (Z = −1.05, *p* = 0.296, r = 0.21), indicating a small effect size. The Hodges–Lehmann median difference was 0.5 (95% CI: −0.5 to 1.5). The mean VAS score for restorations fabricated based on c-CR recordings was 6.6 ± 2.1 (median: 7.0), whereas restorations fabricated based on d-CR recordings had a mean VAS score of 8.0 ± 1.4 (median: 8.0). Although the difference approached statistical significance (Z = −1.95, *p* = 0.051), the effect size was moderate (r = 0.40). The Hodges–Lehmann median difference was 1.5 (95% CI: 0.5 to 3.0). The mean recording time for c-CR recordings was 10.7 ± 2.2 min (median: 10.5), whereas that for d-CR recordings was 20.0 ± 2.2 min (median: 19.5). A statistically significant difference was observed between the two methods (Z = −3.06, *p* = 0.002), with a large effect size (r = 0.63). The Hodges–Lehmann median difference was 9.0 min (95% CI: 6.0 to 12.0). Table 1 summarizes the comparison of patient-reported outcome measures (VAS scores) and recording times between c-CR and d-CR.

The correlation between recording time and VAS scores related to patient-reported experience during the jaw relation recording procedure was evaluated using Spearman’s correlation analysis. No statistically significant correlation was found between recording time and VAS scores for either recording method (c-CR: ρ = −0.160, *p* = 0.619; d-CR: ρ = −0.126, *p* = 0.697). A moderate, positive association was observed between VAS scores related to jaw relation recording experience and VAS scores assigned to the restorations for both methods. In the c-CR group, this association was ρ = 0.577 (*p* = 0.05), whereas in the d-CR group, the association was ρ = 0.539 (*p* = 0.07).

## 4. Discussion

In the present study, the recording of CR with conventional and digital techniques was evaluated in patients requiring occlusal rehabilitation, with a specific focus on patient-reported experience during the jaw relation recording stage and patient-reported comfort during restoration try-in. Overall, the findings indicate that both approaches were perceived similarly by patients across the evaluated clinical stages. The primary null hypothesis is not rejected for either the jaw relation recording stage or the restoration try-in stage. Accordingly, the insignificant difference in patient-reported outcomes suggests that the choice between c-CR and d-CR may not substantially influence patient comfort or procedural experience during either jaw relation recording or restoration try-in.

Recording time alone does not provide information regarding patient perception or the qualitative aspects of the clinical procedure. Therefore, the assessment of jaw relation recording methods should not rely solely on time-based metrics but should also incorporate patient-centered outcome measures to achieve a more comprehensive evaluation of clinical performance.

Correlation analyses demonstrated a moderate, positive association between VAS scores related to jaw relation recording experience and VAS scores assigned to the restorations for both methods. However, as these associations remained at a borderline level of statistical significance and restoration evaluations were performed under blinded conditions, a causal relationship between patient experience during the recording stage and restoration assessment cannot be established.

In addition, the absence of a statistically significant correlation between recording time and patient-reported experience during the jaw relation recording procedure indicates that patient perception cannot be explained solely by quantitative time-based measures. Accordingly, the secondary null hypothesis, stating that no correlation exists between recording time and patient-reported experience, was accepted.

PROMs represent a subjective and multidimensional construct that reflects patient perception, comfort, tolerance, and shared decision-making throughout the treatment process, independent of technical clinical success or isolated quantitative parameters such as procedure time. This aspect is particularly relevant in clinical procedures requiring active patient cooperation, such as jaw relation recording, where patient experience may influence both treatment acceptance and clinical feasibility. Previous studies have emphasized that patient perception cannot be fully explained by measurable time-related parameters alone, as it is shaped by contextual, experiential, and individual factors [27,28,29,32]. The absence of a significant correlation between recording time and patient-reported experience observed in the present study is consistent with these findings.

In the c-CR technique, CR is determined using bimanual manipulation, which may be regarded as a more simplified and clinically accessible approach. Although axiographic systems have been proposed for the functional assessment of mandibular movements during centric relation determination, their clinical application is often considered technique-sensitive and highly dependent on clinician experience, which has limited their widespread adoption in routine clinical practice. Consequently, simplified methods for CR determination remain commonly used.

Recording the mandibular position based on a single static registration may necessitate increased occlusal adjustment during the restorative phase and may additionally be associated with the potential development of occlusal adjustment-related clinical and prosthetic complications. Objective clinical parameters commonly used to evaluate restorative outcomes, including occlusal contact distribution, morphological congruence, and prosthetic acceptability, were beyond the scope of the present study and were therefore not assessed [12,46]. The lack of objective restorative evaluations limits the interpretation of prosthetic outcomes associated with single-position digital-centric relation records. Further clinical studies incorporating objective outcome measures are required to clarify the effects of occlusal adjustment and related prosthetic complications.

In contrast, d-CR requires the use of a jaw-tracking device capable of analyzing mandibular movements, introducing additional clinical considerations related to clinician experience and patient tolerance of the tracking system. Despite this increased technological and procedural complexity, the findings of the present study demonstrate that the d-CR approach does not result in a statistically significant difference in patient-reported outcomes when compared with the c-CR approach. Although statistical significance was not reached for restoration try-in comfort, the observed moderate effect size suggests that the magnitude of the difference may warrant further investigation in larger cohorts. This observation suggests that, despite the additional technological complexity of digitally determined centric relation, the method is well accepted by patients and does not negatively influence patient-reported experience.

Beyond patient-reported outcomes, digitally determined centric relation may offer several potential clinical and technical advantages. Conventional CR techniques rely on interocclusal registration materials that may be affected by deformation or dimensional instability, potentially influencing positional accuracy. The digital approach eliminates this material-dependent step, thereby reducing the risk of such inaccuracies. In addition, the acquisition of multiple mandibular closure recordings enables real-time assessment of positional consistency and allows immediate detection of deviations. The calculation of an averaged mandibular position from repeated recordings may further reduce random variability and enhance reproducibility. Moreover, direct transfer of the recorded mandibular position into CAD-based software facilitates seamless integration into a fully digital workflow.

The level of clinician experience may influence the performance of both techniques. In the present study, all centric relation CR recordings were performed by a prosthodontic specialist with at least two years of focused clinical experience in CR determination, who routinely applies both bimanual manipulation and digitally assisted recording methods in daily practice. Although bimanual manipulation can theoretically be performed by less experienced clinicians, the accuracy and reproducibility of the record may be questionable under such circumstances, given the technique-sensitive and operator-dependent nature of the method. In contrast, digitally determined CR requires appropriate device setup, calibration, and proficient software navigation, necessitating specific training and clinical familiarity. Therefore, this approach also involves a distinct learning curve. Despite these operator-related considerations, no significant differences were observed between the two techniques in terms of PROMs. From a patient-centered perspective, this finding suggests that digitally assisted CR determination may represent a clinically promising alternative, demonstrating comparable patient acceptance to the conventional approach.

With the emergence of digital technologies in dentistry, numerous studies have evaluated digital workflows involving intraoral scanners across different clinical scenarios from the perspective of patient-reported outcome measures (PROMs) [12,28,29,47,48,49,50,51,52]. These studies have highlighted the impact of digital impression techniques on patient comfort, procedural experience, and overall perception of clinical procedures, emphasizing the importance of PROM-based evaluations. Similarly, the increasing clinical use of jaw-tracking devices has generated a growing interest in assessing methods of digital jaw relation recording from a PROM perspective. However, despite this growing interest, no clinical studies have directly compared the recordings of conventionally determined CR and digitally determined CR based on PROMs. In this context, the present study provides a PROM-centered approach by incorporating patient perception into the evaluation of digital jaw relation recording methods within routine clinical practice.

In clinical practice, the selection of a jaw relation recording method should not be based solely on scientific validity or procedural simplicity, but should also consider patient-related factors. c-CR and d-CR may exert variable effects across different stages of clinical application and patient perception. Accordingly, the combined evaluation of PROMs and clinical time parameters may support more rational and individualized planning of digital prosthetic workflows. When patient-reported comfort appears comparable between techniques, factors such as reproducibility, digital integration, and data transfer efficiency may become relevant in clinical decision-making.

Although digitally determined centric relation represents a relatively newer technological approach, the findings of the present study indicate that its clinical applicability is predictable and comparable to conventional methods in terms of patient-reported experience.

Several limitations of the present study should be acknowledged. First, the relatively small sample size (*n* = 12) limits the generalizability of the findings. Although the study achieved acceptable post hoc statistical power, the results should be interpreted as exploratory and hypothesis-generating rather than definitive clinical evidence. Second, objective occlusal parameters and long-term restorative outcomes were not evaluated. Therefore, the findings reflect short-term patient-reported perceptions and may not fully represent long-term functional stability. Future studies with larger cohorts and longitudinal follow-up are required to confirm these results.

In addition, the recording sessions were conducted in a fixed sequence for all participants due to the requirements of the study design and to ensure procedural standardization. While randomization was implemented during the restoration try-in phase, the absence of randomization during the recording phase may have introduced a potential order effect. A formal carryover analysis was not performed, as the primary outcome measure consisted of immediate patient-reported comfort assessments obtained within the same clinical session. Given the short-term and perceptual nature of the evaluated parameters, the biological plausibility of a true carryover effect was considered limited. Nevertheless, the potential influence of sequence-related bias cannot be completely excluded and should be considered when interpreting the results.

## Figures and Tables

**Table 1:** Comparison of Visual Analog Scale (VAS) scores assessing patient-reported experience during jaw relation recording and comfort during try-in and recording time between conventionally determined centric relation (c-CR) and digitally determined centric relation (d-CR).

Outcome	Method	Mean	SD	MD	Min.	Max.	Z	*p*	r
**Jaw Relation Recording**	c-CR	7.7	1.3	7.0	6	10	−1.05	0.296	0.21
	d-CR	7.0	1.7	7.5	5	10			
**Restoration Try-in**	c-CR	6.6	2.1	7.0	2	10	−1.95	0.051	0.40
	d-CR	8.0	1.4	8.0	5	10			
**Recording Time (minute)**	c-CR	10.7	2.2	10.5	8	15	−3.06	0.002 *	0.63
	d-CR	20.0	2.2	19.5	17	25			

(Abbreviations: SD, standard deviation; MD, median; Min., minimum; Max., maximum. * Statistically significant at *p* < 0.05.).

## Data Availability

The data presented in this study are available on request from the corresponding author.

## References

[1] Gross M., Gracis S., Gamborena I., Meyenberg K., Shifman A., Nissan J. (2015). The Science and Art of Occlusion and Oral Rehabilitation.

[2] Alhajj M.N., Khalifa N., Abduo J., Amran A.G., Ismail I.A. (2017). Determination of occlusal vertical dimension for complete dentures patients: An updated review. J. Oral Rehabil..

[3] The Academy of Prosthodontics (2017). The Glossary of Prosthodontic Terms: Ninth Edition. J. Prosthet. Dent..

[4] Manfredini D., Ercoli C., Poggio C.E., Carboncini F., Ferrari M. (2023). Centric relation—A biological perspective of a technical concept. J. Oral Rehabil..

[5] Kois J., Phillips K. (1997). Occlusal vertical dimension: Alteration concerns. Compend. Contin. Educ. Dent. (Jamesburg, N.J. 1995).

[6] Nakamura Y., Hojo S., Sato H. (2010). The effect of surface roughness on the Weibull distribution of porcelain strength. Dent. Mater. J..

[7] Curran P., Cattani-Lorente M., Wiskott H.A., Durual S., Scherrer S.S. (2017). Grinding damage assessment for CAD-CAM restorative materials. Dent. Mater..

[8] Denry I., Kelly J.R. (2008). State of the art of zirconia for dental applications. Dent. Mater..

[9] Zhang Y., Lawn B.R. (2019). Evaluating dental zirconia. Dent. Mater..

[10] Canneto J.-J., Cattani-Lorente M., Durual S., Wiskott A.H., Scherrer S.S. (2016). Grinding damage assessment on four high-strength ceramics. Dent. Mater..

[11] Zarbakhsh A., Jalalian E., Samiei N., Mahgoli M.H., Ghane H.K. (2021). Accuracy of digital impression taking using intraoral scanner versus the conventional technique. Front. Dent..

[12] Karasan D., Sailer I., Lee H., Demir F., Zarauz C., Akca K. (2023). Occlusal adjustment of 3-unit tooth-supported fixed dental prostheses fabricated with complete-digital and-analog workflows: A crossover clinical trial. J. Dent..

[13] Wang J., Wang B., Liu Y., Luo Y., Wu Y., Xiang L., Yang X., Qu Y., Tian T., Man Y. (2024). Recent advances in digital technology in implant dentistry. J. Dent. Res..

[14] Radu M., Radu D., Abboud M. (2020). Digital recording of a conventionally determined centric relation: A technique using an intraoral scanner. J. Prosthet. Dent..

[15] Solaberrieta E., Arias A., Brizuela A., Garikano X., Pradies G. (2016). Determining the requirements, section quantity, and dimension of the virtual occlusal record. J. Prosthet. Dent..

[16] Park J.H., Lee G.-H., Moon D.-N., Kim J.-C., Park M., Lee K.-M. (2021). A digital approach to the evaluation of mandibular position by using a virtual articulator. J. Prosthet. Dent..

[17] Neto C.L.D.M.M., Dos Santos D.M., de Magalhães Bertoz A.P., de Melo Moreno A.L., Goiato M.C. (2021). Comparison of techniques for obtaining centric relation based on the reproducibility of the condylar positions in centric relation—A systematic review. Eur. J. Dent..

[18] Hellmann D., Becker G., Fingerhut C., Schmitter M., Rammelsberg P., Schindler H.J. (2014). Methods of determining centric relation: A comparative study. J. Cranio-Mand. Func..

[19] Dawson P.E. (2006). Functional Occlusion: From TMJ to Smile Design.

[20] Dawson P.E. (1995). New definition for relating occlusion to varying conditions of the temporomandibular joint. J. Prosthet. Dent..

[21] McKee J.R. (1997). Comparing condylar position repeatability for standardized versus nonstandardized methods of achieving centric relation. J. Prosthet. Dent..

[22] Theusner J., Plesh O., Curtis D.A., Hutton J.E. (1993). Axiographic tracings of temporomandibular joint movements. J. Prosthet. Dent..

[23] Posselt U. (1962). Physiology of Occlusion and Rehabilitation.

[24] Parlett K., Paesani D., Tallents R., Hatala M. (1993). Temporomandibular joint axiography and MRI findings: A comparative study. J. Prosthet. Dent..

[25] Piehslinger E., Celar A., Celar R., Jäger W., Slavicek R. (1993). Reproducibility of the condylar reference position. J. Orofac. Pain.

[26] Wittneben J., Wismeijer D., Brägger U., Joda T., Abou-Ayash S. (2018). Patient-reported outcome measures focusing on aesthetics of implant-and tooth-supported fixed dental prostheses: A systematic review and meta-analysis. Clin. Oral Implant. Res..

[27] Yuzbasioglu E., Kurt H., Turunc R., Bilir H. (2014). Comparison of digital and conventional impression techniques: Evaluation of patients’ perception, treatment comfort, effectiveness and clinical outcomes. BMC Oral Health.

[28] Gjelvold B., Chrcanovic B.R., Korduner E., Collin-Bagewitz I., Kisch J. (2016). Intraoral digital impression technique compared to conventional impression technique. A randomized clinical trial. J. Prosthodont..

[29] Pachiou A., Zervou E., Sykaras N., Tortopidis D., Ioannidis A., Jung R.E., Strauss F.J., Kourtis S. (2025). Patient-Reported Outcomes of Digital Versus Conventional Impressions for Implant-Supported Fixed Dental Prostheses: A Systematic Review and Meta-Analysis. J. Pers. Med..

[30] Jokstad A. (2018). Patient-reported outcomes (PROs) versus patient-reported outcome measures (PROMs)—Is there a difference?. Clin. Exp. Dent. Res..

[31] Hua F. (2023). Dental patient-reported outcomes update 2022. J. Evid.-Based Dent. Pract..

[32] Hua F. (2025). Dental Patient-Reported Outcomes Update 2024. J. Evid.-Based Dent. Pract..

[33] Reissmann D.R. (2019). Dental Patient-Reported Outcome Measures Are Essential for Evidence-Based Prosthetic Dentistry.

[34] Aiyegbusi O.L., Rivera S.C., Roydhouse J., Kamudoni P., Alder Y., Anderson N., Baldwin R.M., Bhatnagar V., Black J., Bottomley A. (2024). Recommendations to address respondent burden associated with patient-reported outcome assessment. Nat. Med..

[35] Lamont T.J., Clarkson J.E. (2022). Core outcome sets and dental patient reported outcomes. J. Evid.-Based Dent. Pract..

[36] Hughes S.E., Anderson N.E., Hathaway E., McMullan C., Hughes B.M.A., Collis P., Peipert J.D., Haroon S., Calvert M. (2024). Opportunities and challenges for patient-reported outcome assessment in multimorbidity research and practice. Nat. Med..

[37] Chander N.G. (2019). Visual Analog Scale in Prosthodontics.

[38] Mariotti A., Hefti A.F. (2015). Defining periodontal health. BMC Oral Health.

[39] Schiffman E., Ohrbach R., Truelove E., Look J., Anderson G., Goulet J.P., List T., Svensson P., Gonzalez Y., Lobbezoo F. (2014). Diagnostic criteria for temporomandibular disorders (DC/TMD) for clinical and research applications: Recommendations of the International RDC/TMD Consortium Network and Orofacial Pain Special Interest Group. J. Oral Facial Pain Headache.

[40] Mühlemann H.R. (1967). Tooth mobility: A review of clinical aspects and research findings. J. Periodontol..

[41] Hohmann A., Hielscher W. (2014). Foundations of Dental Technology: Anatomy and Physiology.

[42] Rosenstiel S.F., Land M., Fujimoto J. (2006). Contemporary Fixed Prosthodontics.

[43] Goodacre C.J., Campagni W.V., Aquilino S.A. (2001). Tooth preparations for complete crowns: An art form based on scientific principles. J. Prosthet. Dent..

[44] Bumann A., Lotzmann U. (2002). TMJ Disorders and Orofacial Pain: The Role of Dentistry in a Multidisciplinary Diagnostic Approach.

[45] Win T.T., Mai H.-N., Kim S.-Y., Cho S.-H., Kim J.-E., Srimaneepong V., Kaenploy J., Lee D.-H. (2025). Fit accuracy of complete crowns fabricated by generative artificial intelligence design: A comparative clinical study. J. Adv. Prosthodont..

[46] Zhang Y., Tian J., Wei D., Di P., Lin Y. (2019). Quantitative clinical adjustment analysis of posterior single implant crown in a chairside digital workflow: A randomized controlled trial. Clin. Oral Implant. Res..

[47] Joda T., Zarone F., Ferrari M. (2017). The complete digital workflow in fixed prosthodontics: A systematic review. BMC Oral Health.

[48] Mühlemann S., Kraus R.D., Hämmerle C.H.F., Thoma D.S. (2018). Is the use of digital technologies for the fabrication of implant-supported reconstructions more efficient and/or more effective than conventional techniques: A systematic review. Clin. Oral Implant. Res..

[49] Bishti S., Tuna T., Rittich A., Wolfart S. (2021). Patient-reported outcome measures (PROMs) of implant-supported reconstructions using digital workflows: A systematic review and meta-analysis. Clin. Oral Implant. Res..

[50] Bandiaky O.N., Le Bars P., Gaudin A., Hardouin J.B., Cheraud-Carpentier M., Mbodj E.B., Soueidan A. (2022). Comparative assessment of complete-coverage, fixed tooth-supported prostheses fabricated from digital scans or conventional impressions: A systematic review and meta-analysis. J. Prosthet. Dent..

[51] Haddadi Y., Bahrami G., Isidor F. (2018). Evaluation of operating time and patient perception using conventional impression taking and intraoral scanning for crown manufacture: A split-mouth, randomized clinical study. Int. J. Prosthodont..

[52] Sakornwimon N., Leevailoj C. (2017). Clinical marginal fit of zirconia crowns and patients’ preferences for impression techniques using intraoral digital scanner versus polyvinyl siloxane material. J. Prosthet. Dent..

